# Sleep Problems and Health Outcomes Among Urban American Indian and Alaska Native Adolescents

**DOI:** 10.1001/jamanetworkopen.2024.14735

**Published:** 2024-06-04

**Authors:** Wendy M. Troxel, David J. Klein, Lu Dong, Zahra Mousavi, Daniel L. Dickerson, Carrie L. Johnson, Alina I. Palimaru, Ryan A. Brown, Anthony Rodriguez, Jennifer Parker, Kurt Schweigman, Elizabeth J. D’Amico

**Affiliations:** 1 Behavioral and Policy Sciences, Division of Social and Economic Wellbeing, RAND Corporation, Pittsburgh, Pennsylvania; 2 Behavioral and Policy Sciences, Division of Social and Economic Wellbeing, RAND Corporation, Santa Monica, California; 3 UCLA Integrated Substance Abuse Program, Semel Institute for Neuroscience and Human Behavior, Los Angeles, California; 4 Sacred Path Indigenous Wellness Center, Los Angeles, California; 5 Behavioral and Policy Sciences, Division of Social and Economic Wellbeing, RAND Corporation, Boston, Massachusetts; 6 Public Health Consultant, Santa Rosa, California

## Abstract

**Importance:**

Adolescent sleep problems are prevalent, particularly among racial and ethnic minority groups, and can increase morbidity. Despite the numerous strengths of their racial and ethnic group, urban American Indian and Alaska Native adolescents face significant health disparities but are rarely included in health research. Understanding how sleep problems are associated with health outcomes among American Indian and Alaska Native adolescents may elucidate novel targets for interventions to promote health equity.

**Objective:**

To assess whether baseline sleep problems are associated with changes in behavioral and cardiometabolic health outcomes among urban American Indian and Alaska Native adolescents 2 years later.

**Design, Setting, and Participants:**

American Indian and Alaska Native adolescents were recruited via flyers and community events for an observational cohort study in California. Baseline assessments were conducted among 142 adolescents from March 1, 2018, to March 31, 2020, and follow-ups were conducted among 114 adolescents from December 1, 2020, to June 30, 2022.

**Exposures:**

Baseline actigraphy-assessed sleep duration and efficiency and self-reported sleep disturbances and social jet lag (absolute value of the difference in sleep midpoint on weekends vs weekdays; indicator of circadian misalignment).

**Main Outcomes and Measures:**

Main outcome measures included self-reported depression (measured using the Patient Health Questionnaire), anxiety (measured using the Generalized Anxiety Disorder 7-item scale), past year alcohol and cannabis use, body mass index, systolic blood pressure (SBP) and diastolic blood pressure (DBP), waist circumference, and glycosylated hemoglobin (HbA_1c_). Analyses examined whether baseline sleep was associated with health outcomes at follow-up, controlling for age, sex, and baseline outcome measures.

**Results:**

The baseline sample included 142 urban American Indian and Alaska Native adolescents (mean [SD] age, 14.0 [1.4] years; 84 girls [59%]), 80% of whom (n = 114; mean [SD] age, 14.1 [1.3] years; 71 girls [62%]) completed follow-ups. Linear or logistic regressions showed significant negative associations between shorter sleep duration and depression (β = −1.21 [95% CI, −2.19 to −0.24]), anxiety (β = −0.89 [95% CI, −1.76 to −0.03]), DBP (β = −2.03 [95% CI, −3.79 to −0.28]), and HbA_1c_ level (β = −0.15 [95% CI, −0.26 to −0.04]) and likelihood of alcohol (odds ratio [OR], 0.57 [95% CI, 0.36-0.91]) and cannabis use (full week: OR, 0.59 [95% CI, 0.35-0.99]) at follow-up. Greater social jet lag was associated with significantly higher SBP (β = 0.06 [95% CI, 0.01-0.11]) at follow-up.

**Conclusions and Relevance:**

This cohort study found significant associations between poor sleep and adverse changes in health outcomes. Findings highlight the importance of developing culturally responsive interventions that target sleep as a key modifiable risk factor to improve the health of American Indian and Alaska Native adolescents.

## Key Points

**Question:**

Is poor sleep associated with adverse changes in behavioral and cardiometabolic health outcomes among urban American Indian and Alaska Native adolescents?

**Findings:**

In this cohort study of 142 urban American Indian and Alaska Native adolescents, shorter sleep duration was associated with increases in depression and anxiety symptoms, likelihood of alcohol and cannabis use, diastolic blood pressure, and glycosylated hemoglobin at 2-year follow-up. Greater social jet lag was associated with significant increases in systolic blood pressure at follow-up.

**Meaning:**

This study suggests that interventions that target sleep may be a novel approach to promoting health equity among American Indian and Alaska Native adolescents, a population that faces significant health disparities but has rarely been included in research.

## Teaser

This cohort study assesses whether baseline sleep problems are associated with adverse changes in behavioral and cardiometabolic health outcomes among urban American Indian and Alaska Native adolescents 2 years later.

## Introduction

American Indian and Alaska Native people have significant strengths, including strong cultural identity and shared values and traditions, which serve as protective health factors.^1,2,3,4^ However, the legacy and enduring effects of historically based trauma, such as forced relocation from tribal homelands, have contributed to disproportionately higher chronic disease burden among this population, including increased risk for alcohol and other drug use,^5,6^ suicide,^7,8^ obesity,^9^ type 2 diabetes,^10^ and cardiovascular disease.^11,12,13^ The COVID-19 pandemic exposed important sources of resilience in American Indian and Alaska Native people, including community connection and family cohesion,^14^ but also highlighted inequities, including increased exposure to poverty, crowding, food insecurity, and reduced health care access, which increase susceptibility to COVID-19 infection and mortality.^15,16^ Thus, it is critical to consider how health and social determinants are jointly associated with health disparities among American Indian and Alaska Native people.

Most health disparities work within American Indian and Alaska Native people has focused on adults and those living on reservations or tribal lands.^17^ To identify prevention and intervention opportunities, it is important to examine the emergence of health behaviors earlier in life, such as adolescence.^18,19^ Sleep problems, including insomnia, short sleep duration, fragmented sleep, and circadian rhythm disruptions, are highly prevalent among general adolescent populations^20,21^ and are modifiable risk factors for cardiometabolic, mental, and behavioral health outcomes.^22,23,24,25,26^ Existing work on sleep and cardiometabolic risk among adolescents is predominantly cross-sectional and based on self-reported sleep,^27,28^ which is subject to bias.^29,30^ Moreover, although American Indian and Alaska Native youths are more likely to engage in risky health behaviors compared with their peers who are not American Indian and Alaska Native,^31^ to our knowledge, there has been no prior study of how sleep is associated with health changes over time among American Indian and Alaska Native adolescents.^32^

Structurally racist policies, such as forced relocations and mandatory boarding schools, sought to dismantle American Indian and Alaska Native communities and culture.^33,34^ Thus, approximately 80% of American Indian and Alaska Native people currently live off tribal lands, many in urban environments, yet they rarely are included in research.^35^ These individuals face a confluence of risk factors, including exposure to poverty, crowding, limited access to health-promoting resources, and discrimination, as well as reduced connection to American Indian and Alaska Native community and cultural resources that can otherwise serve as protective factors.^17,36^ For example, cross-sectional analyses from the current cohort demonstrated significant correlations between greater food insecurity and poorer sleep and cardiometabolic health.^37^

This analysis expands our prior work^37^ and the literature in general by examining the degree to which objectively and subjectively measured sleep problems are associated with changes in mental, behavioral, and cardiometabolic health outcomes 2 years later in a sample of urban American Indian and Alaska Native adolescents. We hypothesized that greater self-reported sleep disturbance, social jet lag (absolute value of the difference in sleep midpoint on weekends vs weekdays; indicator of circadian misalignment), and actigraphy-assessed shorter sleep duration and poorer (ie, lower) sleep efficiency would be associated with worse mental, behavioral, and cardiometabolic health over a 2-year follow-up period.

## Methods

### Participants and Procedures

Eligible participants comprised 142 American Indian and Alaska Native adolescents residing in urban areas in California who completed a baseline assessment from March 1, 2018, to March 31, 2020, for the Native American Youth Sleep Health and Wellness study. The analytic sample includes 114 adolescents between 12 and 16 years of age who completed the baseline survey and a follow-up assessment approximately 2 years later (December 1, 2020, to June 30, 2022). Given known variability in adolescent sleep-wake schedules between the school year and summer, all data collection efforts occurred during the school year (late August to June) and during a “typical week,” when participants did not report illness (COVID-19 or otherwise). Participants self-identified or were identified by family as American Indian and Alaska Native. Adolescents with major neurologic conditions (including intellectual disability), chronic medical conditions (eg, cancer, diabetes, cardiovascular disease), diagnosis of sleep apnea or restless legs syndrome, and pregnant adolescents were excluded. Demographics and sleep measures were collected at baseline. Outcome measures were assessed at baseline and follow-up. RAND’s internal review board and the study Elder Advisory Board approved all procedures. Parents provided written consent and adolescents provided assent. This report followed the Strengthening the Reporting of Observational Studies in Epidemiology (STROBE) reporting guideline.

Baseline data were collected during a home visit (surveys, anthropometric measures, and blood draw) and during a 7-day sleep diary and actigraphy period. The COVID-19 pandemic delayed fielding of follow-up assessments as we were unable to perform in-person data collection (required for cardiometabolic measures) until the statewide mandatory stay-at-home period was lifted (June 2021) and our American Indian and Alaska Native community advisory board approved resuming in-person research activities (July 2021). To avoid delaying all data collection, in December 2020 we began fielding the follow-up survey online. Thus, for participants who completed the web survey prior to July 2021 (n = 75), there was a time delay between the survey and cardiometabolic data collection. Furthermore, we were not able to recontact all participants. Analyses involving anthropometric measures at follow-up are restricted to 97 participants with available data.

Consistent with best practices,^38,39,40^ we used a community-based participatory approach to engage American Indian and Alaska Native communities. Our collaborator, Sacred Path Indigenous Wellness Center, ensured that all recruitment and assessment were culturally appropriate, and one of the principal investigators on the project is Native American with more than 2 decades of experience. For example, we pilot tested all materials with community members, created a study logo based on community input, hosted community meetings to address questions, hired American Indian and Alaska Native recruiters, and conducted cultural awareness training for staff (eAppendix in Supplement 1).

### Measures

#### Demographics

Participants reported their age, sex, race and ethnicity, school grade, tribal affiliation, and mother’s educational level.

#### Self-Reported Sleep Measures

We measured sleep disturbances using the validated sleep-wake problem subscale from the School Sleep Habits Survey for Adolescents,^41^ which focuses on the frequency of indicators of erratic sleep or wake behaviors over the past 2 weeks (eg, stayed awake past 3:00 am or later). Items were rated on a scale of 1 (never) to 5 (every day or night); the sum of the 15 items ranged from 15 to 75 (observed range, 15-58). Higher scores indicate greater sleep disturbances (α = 0.80).

Social jet lag was calculated as the absolute value of the difference in the midpoint of sleep (ie, middle time point between diary-reported bedtimes and wake times) between weekdays and weekends, expressed in minutes (sample range, 6-238 minutes). Higher values indicate a greater degree of circadian misalignment.^42^

#### Objective Sleep Measures

Participants wore the Phillips Respironics Actigraph-2, a lightweight, waterproof wrist-worn accelerometer for approximately 7 days (mean [SD] wear time, 7.2 [1.1] days). Actigraphs recorded in 30-second epochs at medium sensitivity. Sleep diary–reported bedtimes and wake times were used to set rest intervals for deriving sleep parameters using the Actiware program–validated scoring algorithms.^43^ Actigraphy sleep measures were averaged across days and included sleep duration (time in bed minus sleep onset latency minus wake after sleep onset minus terminal wakefulness) and sleep efficiency (percentage of the time in bed spent asleep; higher scores indicate more consolidated sleep; range, 55%-93%). Given known variability in adolescent sleep duration on weekends vs weekdays,^20^ we analyzed sleep duration based on both full-week data and weekday-only data. Sleep duration ranged from 4.6 to 9.1 hours per night for the full week and 3.5 to 9.1 hours per night on weekdays.

### Mental and Behavioral Health

#### Mental Health Symptoms

At baseline, depressive symptoms were measured using the 2-item Patient Health Questionnaire screening tool (PHQ-2), validated in adolescent samples as a screening tool for major depression.^44^ At follow-up, depressive symptoms were assessed using the 8-item Patient Health Questionnaire screening tool (PHQ-8). The PHQ measures are sums of items scored from 0 (not at all) to 3 (nearly every day), so the PHQ-2 score ranges from 0 to 6, while the PHQ-8 has a score range of 0 to 24, with higher scores indicating more depression. Anxiety symptoms were measured using the Generalized Anxiety Disorder 7-item scale (GAD-7),^45^ also validated in adolescents. The GAD-7 also sums items scored 0 to 3 and scores range from 0 to 21, with higher scores indicating more anxiety.

#### Substance Use

Participants reported past-year use of alcohol or cannabis with items from the Monitoring the Future survey.^46^ Due to low frequency of use at baseline, alcohol or cannabis use was coded as any use vs no past-year use.

### Cardiometabolic Health

#### Anthropometric Measures

Waist circumference (baseline range, 62-123 cm) was measured at the level of the umbilicus to provide a measure of central adiposity. Body mass index (BMI [calculated as weight in kilograms divided by height in meters squared]; baseline range, 14.8-47.3) was measured using a digital scale to assess weight and a stadiometer to assess height. Standardized BMI was derived using age and sex norms from the Centers for Disease Control and Prevention Growth Charts.^47^

#### Blood Pressure

Measurements of resting blood pressure were collected by a certified reader using clinically validated blood pressure monitors (Omron HEM-907XL professional blood pressure monitors). Up to 4 consecutive measurements were taken until 2 yielded differences of less than 0.5 for both systolic blood pressure (SBP) and diastolic blood pressure (DBP). We used the mean of those 2 measurements. At baseline, SBP ranged from 92 to 146 mm Hg and DBP ranged from 50 to 88 mm Hg.

#### Glycosylated Hemoglobin

Based on feedback from our advisory board and to reduce participant burden, we selected glycosylated hemoglobin (HbA_1c_) as a stable measure of blood glucose that can be reliably measured with a nonfasting blood sample.^48^ A trained phlebotomist obtained the blood samples from the antecubital vein during the home visit. Assays for HbA_1c_ were performed by Quest Diagnostics. At baseline, 10 participants refused the blood draw, and 1 sample could not be assayed. At follow-up, of the 97 participants who completed anthropometric measures, 87 had blood data available and assayed for HbA_1c_ (range, 4.6-6.5 mmol/L); 10 were missing data due to participant refusal or technical issues with the assay.

### Statistical Analysis

Independent variables (ie, sleep measures) were entered as continuous variables into models. We used multiple linear regression for continuous outcomes or logistic regression models for binary outcomes (ie, alcohol and cannabis use) in SAS, version 9.4 (SAS Institute Inc), to examine whether baseline sleep measures were associated with changes in mental, behavioral, or cardiometabolic health outcomes at follow-up, controlling for baseline measures of the given outcome. Models also controlled for participants’ sex, age, and maternal educational level. All statistical tests were 2-sided, and *P* < .05 was determined a priori to indicate statistical significance. A total of 45 models were run (5 sleep variables and 9 outcomes).

We performed complete-case analysis on outcomes and primary independent variables. Age and sex were never missing. Maternal educational level was missing for 12 participants, mainly due to “don’t know” responses; this was addressed with multiple imputation. The 25 imputed datasets were analyzed with standard methods, and results were combined with SAS Proc MIANALYZE, version 9.4 (SAS Institute Inc).

## Results

Analyses comparing demographic characteristics of the full baseline sample (N = 142; mean [SD] age, 14.0 [1.4] years; 84 girls [59%] and 58 boys [41%]; 73 Hispanic adolescents [51%]) with the analytic sample who (1) completed sleep and survey assessments at baseline and follow-up (n = 114; mean [SD] age, 14.1 [1.3] years; 71 girls [62%] and 43 boys [38%]; 56 Hispanic adolescents [49%]) or (2) completed baseline and follow-up anthropometric measures (n = 97; mean [SD] age, 14.0 [1.3] years; 61 girls [63%] and 36 boys [37%]; 51 Hispanic adolescents [53%]) revealed no significant differences in demographic characteristics (Table 1). There were also no demographic differences between the sample with follow-up survey data (n = 114) and those with follow-up anthropometric data (n = 97). Just over half the sample reported maternal educational level as some college or a college degree. More than 40 tribes were represented (tribal affiliation is not reported to protect confidentiality).^49^

**Table 1.** Baseline Demographic Characteristics of the Sample

Characteristic	Analytic sample (n = 114)	Baseline sample (N = 142)	Anthropometric sample (n = 97)^a^
Sex assigned at birth, No. (%)			
Male	43 (38)	58 (41)	36 (37)
Female	71 (62)	84 (59)	61 (63)
Hispanic, No. (%)			
Yes	56 (49)	73 (51)	51 (53)
No	58 (51)	69 (49)	46 (47)
Current grade, No. (%)			
6th	3 (3)	4 (3)	2 (2)
7th	19 (17)	25 (18)	17 (18)
8th	26 (23)	36 (25)	22 (23)
9th	32 (28)	35 (25)	29 (30)
10th	16 (14)	21 (15)	11 (11)
11th	18 (16)	21 (15)	16 (16)
Age (continuous), mean (SD), y	14.1 (1.3)	14.0 (1.4)	14.0 (1.3)
Mother’s educational level, No./total No. (%)			
Did not finish high school	12/102 (12)	18/126 (14)	11/85 (13)
Graduated from high school	31/102 (30)	40/126 (32)	23/85 (27)
Some college	26/102 (25)	30/126 (24)	25/85 (29)
Graduated from college	33/102 (32)	38/126 (30)	26/85 (31)

^a^ Sample with anthropometric data available at baseline and follow-up.

Table 2 presents descriptive statistics for baseline sleep measures, as well as baseline and follow-up outcomes. The mean (SD) self-reported sleep disturbance score was 30.6 (8.8). The mean (SD) social jet lag was approximately 84.0 minutes (46.6 minutes), indicating a delay in sleep phase during the weekends relative to weekdays. The mean (SD) actigraphy-measured sleep duration was 7.1 (0.9) hours across the full week and 6.9 (1.0) hours on the weekdays. The mean (SD) actigraphy-measured sleep efficiency was 81.2% (7.0%).

**Table 2.** Descriptive Statistics of Sleep Measures and Mental or Behavioral and Cardiometabolic Health Outcomes

Variables	Baseline	Follow-up
**Sleep (n = 114)**		
Disturbance score, mean (SD)^a^	30.6 (8.8)	NA
Duration (full week), mean (SD), h	7.1 (0.9)	
Duration (weekdays), mean (SD), h	6.9 (1.0)	
Efficiency, mean (SD), %	81.2 (7.0)	
Social jet lag, mean (SD), min	84.0 (46.6)	
**Mental or behavioral health (n = 114)**		
Depression score, mean (SD)^b^	1.0 (1.4)	6.8 (5.5)
Anxiety score, mean (SD)	4.6 (5.1)	6.1 (5.0)
Past-year alcohol use, No. (%)	16 (14)	33 (29)
Past-year cannabis use, No. (%)	22 (19)	34 (30)
**Cardiometabolic health (n = 96)**		
BMI, mean (SD)	24.6 (6.7)	25.3 (6.9)
BMI *z* score, mean (SD)^c^	0.8 (1.3)	0.6 (1.3)
Waist circumference, mean (SD), cm	86.1 (13.7)	87.0 (14.5)
SBP, mean (SD), mm Hg	110.1 (9.6)	115.0 (12.4)
DBP, mean (SD), mm Hg	66.5 (8.3)	70.2 (8.0)
HbA_1c_, mean (SD), mmol/L^d^	5.2 (0.3)	5.2 (0.7)

Abbreviations: BMI, body mass index (calculated as weight in kilograms divided by height in meters squared); DBP, diastolic blood pressure; HbA_1c_, glycosylated hemoglobin; NA, not applicable; PHQ-2, 2-item Patient Health Questionnaire screening tool; PHQ-8, 8-item Patient Health Questionnaire screening tool; SBP, systolic blood pressure.

^a^ Items were rated on a scale of 1 (never) to 5 (every day or night); the sum of the 15 items ranged from 15 to 75 (observed range, 15-58).

^b^ At baseline, depressive symptoms were measured with the PHQ-2 and at follow-up with the PHQ-8. At baseline, 13.3% of participants (15 of 113) met the threshold for clinical depression, while at follow-up, 28.1% of participants (32 of 114) were above the clinical threshold.

^c^ BMI was standardized as a *z* score statistic relative to the distribution of BMI within age and sex based on Centers for Disease Control and Prevention growth charts.

^d^ For HbA_1c_ at baseline, 87 participants; at follow-up, 82 participants.

Depression and anxiety symptoms were low to moderate at both time points. Past-year alcohol and cannabis use was relatively infrequent at baseline (any alcohol use, 16 of 114 [14%]; any cannabis use, 22 of 114 [19%]) but increased to nearly 30% of the sample for both substances at follow-up (any alcohol use, 33 of 114 [29%]; any cannabis use, 34 of 114 [30%]) (Table 2).

Consistent with prior work demonstrating high rates of overweight and obesity as well as central adiposity among American Indian and Alaska Native youths,^9,13^ the mean (SD) BMI was 24.6 (6.7), and the mean (SD) waist circumference was 86.1 (13.7) cm (Table 2). In this sample, 31% of participants (35 of 113) were above the 95th percentile for BMI based on respective age and sex norms. The mean (SD) baseline value of SBP was 110.1 (9.6) mm Hg and of DBP was 66.5 (8.3) mm Hg and was approximately 5 mm Hg higher at follow-up for both measures (SBP, 115.0 [12.4] mm Hg; DBP, 70.2 [8.0] mm Hg). At baseline, average HbA_1c_ was 5.2 (0.3) mmol/L, which remained relatively stable at follow-up (5.2 [0.7] mmol/L).

Table 3 presents results for statistical models of the association of sleep measures at baseline with mental, behavioral, and cardiometabolic health outcomes 2 years later. Shorter sleep duration on weekdays was significantly associated with greater depression symptoms (β = −1.21 [95% CI, −2.19 to −0.24]) and anxiety symptoms (β = −0.89 [95% CI, −1.76 to −0.03]) at follow-up. Shorter sleep duration (both full week and weekdays only) was significantly associated with greater likelihood of cannabis use at follow-up (full week: odds ratio [OR], 0.59 [95% CI, 0.35-0.99]). Shorter weekday sleep duration was significantly associated with a greater likelihood of alcohol use at follow-up (OR, 0.57 [95% CI, 0.36-0.91]). No other sleep measures were significantly associated with follow-up mental or behavioral health outcomes.

**Table 3.** Association Between Baseline Sleep Measures and Mental, Behavioral, and Cardiometabolic Health Outcomes at Follow-Up^a^

Sleep Measure	β (95% CI)							OR (95% CI)	
	Depression	Anxiety	BMI	Waist	SBP	DBP	HbA_1c_	Alcohol use	Cannabis use
Disturbance^b^	0.08 (−0.06 to 0.22)	0.05 (−0.07 to 0.17)	−0.02 (−0.04 to −0.01)	−0.21 (−0.41 to 0.00)	0.06 (−0.22 to 0.33)	0.06 (−0.13 to 0.25)	0.00 (−0.01 to 0.01)	1.00 (0.94 to 1.06)	1.02 (0.96 to 1.08)
Duration (full week), min	−1.03 (−2.14 to 0.08)	−0.70 (−1.68 to 0.28)	−0.04 (−0.20 to 0.11)	0.83 (−1.17 to 2.83)	−1.27 (−3.82 to 1.28)	−2.03 (−3.79 to −0.28)	−0.15 (−0.26 to −0.04)	0.60 (0.36 to 1.02)	0.59 (0.35 to 0.99)
Duration (weekday), min	−1.21 (−2.19 to −0.24)	−0.89 (−1.76 to −0.03)	−0.03 (−0.16 to 0.11)	1.34 (−0.45 to 3.13)	−1.40 (−3.69 to 0.90)	−1.44 (−3.01 to 0.14)	−0.16 (−0.25 to −0.06)	0.57 (0.36 to 0.91)	0.57 (0.36 to 0.90)
Efficiency, %	0.05 (−0.10 to 0.20)	0.09 (−0.04 to 0.22)	−0.01 (−0.03 to 0.01)	0.21 (−0.06 to 0.49)	−0.16 (−0.51 to 0.20)	−0.15 (−0.40 to 0.10)	0.00 (−0.01 to 0.02)	1.02 (0.95 to 1.09)	1.03 (0.96 to 1.11)
Social jet lag, min	0.01 (−0.01 to 0.03)	0.01 (−0.01 to 0.03)	−0.00 (−0.00 to 0.00)	−0.02 (−0.06 to 0.02)	0.06 (0.01 to 0.11)	0.02 (−0.01 to 0.06)	0.00 (−0.00 to 0.00)	1.00 (0.99 to 1.01)	1.00 (0.99 to 1.01)

Abbreviations: BMI, body mass index (calculated as weight in kilograms divided by height in meters squared); DBP, diastolic blood pressure; HbA_1c_, glycosylated hemoglobin; OR, odds ratio; PHQ-2, 2-item Patient Health Questionnaire screening tool; PHQ-8, 8-item Patient Health Questionnaire screening tool; SBP, systolic blood pressure.

^a^ Regression coefficients presented are adjusted for age, sex, maternal educational level, and baseline value of the measure. Body mass index was standardized as a *z* score statistic relative to the distribution of BMI within age and sex based on Centers for Disease Control and Prevention growth charts. For depressive symptoms, baseline value is PHQ-2 (outcome is PHQ-8). Alcohol and cannabis use are binary outcomes coded as any use vs no past-year use.

^b^ Sleep disturbance is the sum of 15 items with response options ranging from 1 = never to 5 = every day or night.

Greater self-reported sleep disturbances at baseline were significantly associated with lower BMI (β = −0.02 [95% CI, −0.04 to −0.01]) and waist circumference (β = −0.21 [95% CI, −0.41 to 0.00]) at follow-up. Greater social jet lag was significantly associated with higher SBP at follow-up (β = 0.06 [95% CI, 0.01-0.11]), whereas shorter sleep duration (full week) was significantly associated with higher DBP (β = −2.03 [95% CI, −3.79 to −0.28]) (Table 3). Finally, shorter sleep duration (full week and weekdays only) was significantly associated with greater HbA_1c_ (full week: β = −0.15 [95% CI, −0.26 to −0.04]).

## Discussion

Urban American Indian and Alaska Native adolescents are a population that is rarely included in research, despite facing a disproportionate burden of chronic health conditions.^4,50^ It is important to understand how sleep may be associated with health disparities among American Indian and Alaska Native adolescents to help inform prevention and intervention programming to increase health equity for this population.

Consistent with prior literature in other adolescent populations,^20^ we found high rates of insufficient sleep, sleep disturbances, social jet lag, and poor sleep efficiency in this urban American Indian and Alaska Native population. For example, mean sleep efficiency in our study was approximately 81%, whereas 85% is commonly used as a clinical cutoff for poor sleep in insomnia treatment studies.^51^ Furthermore, actigraphy-assessed sleep duration was less than 7 hours during the school week, which is far short of current sleep recommendations for adolescents.^52^ To our knowledge, this is the first study to objectively measure sleep among American Indian and Alaska Native adolescents; however, limited prior work using self-reported sleep has also documented high rates of insufficient sleep among American Indian and Alaska Native adolescents.^32,53^

This study also adds to existing literature on how sleep is associated with important health outcomes among American Indian and Alaska Native adolescents.^22,25^ Sleep duration has been the most consistently studied sleep measure and has shown the most robust associations with cardiometabolic outcomes.^27,54^ Consistent with previous work among general adolescent samples and American Indian and Alaska Native adults,^55^ short sleep duration was associated with increases in depression, anxiety, likelihood of alcohol or cannabis use, DBP, and HbA_1c_. Findings support policy initiatives to delay school start times, which have been identified as a factor associated with insufficient sleep among adolescents.^20,56^

Our findings also add to the limited but emerging literature on indices of circadian misalignment, in this case measured as social jet lag and cardiometabolic outcomes.^26,57^ In the current sample, mean social jet lag was 1 hour and 24 minutes, indicating a significant discrepancy in weekday and weekend sleep-wake patterns. Such discrepancies are partly caused by the conflict between adolescents’ biologically based circadian phase delay and early school start times.^20^ We found that greater social jet lag was associated with greater increases in DBP. Although mechanisms underlying this association are not fully understood, adolescents with greater preferences toward eveningness (and therefore greater likelihood of experiencing social jet lag) tend to have shorter, more irregular, and disturbed sleep.^58^ They are also at greater risk for behaviors that can influence blood pressure, including poor diet, physical activity, substance use, and nighttime electronic media use.^24,59,60,61,62^ Unexpectedly, greater self-reported sleep disturbances were associated with lower BMI and waist circumference. This finding contrasts with prior longitudinal work demonstrating that sleep problems are associated with higher BMI, although most of this work has focused on short sleep duration.^63^

Combined, our findings highlight the importance of focusing on multiple sleep parameters for which different interventions may be warranted and emphasize the value of considering multiple health domains potentially associated with sleep. Given that American Indian and Alaska Native youths are disproportionately burdened by mental and behavioral health problems, including depression, suicide, and substance use, as well as increased risk for cardiometabolic disease, these findings support treatment approaches that target sleep as a transdiagnostic process (ie, common pathway) linked with multiple, intersecting domains of health.^64,65^

### Strengths and Limitations

This study’s strengths, including the focus on urban American Indian and Alaska Native adolescents and understanding how sleep is associated with health outcomes and disparities, can help identify opportunities for prevention and intervention for this population.^66,67^ Due to historic abuses of American Indian and Alaska Native people in the name of research,^68^ there is considerable and warranted scientific mistrust. Our team successfully engaged urban American Indian and Alaska Native youths and their caregivers, conducting thorough health assessments, demonstrating our dedication to community-based research and partnerships in urban American Indian and Alaska Native communities.

This study also has some limitations. First, we focused specifically on American Indian and Alaska Native adolescents from urban areas in southern, central, and northern California. Thus, findings may not be generalized to other racial and ethnic groups or to urban American Indian and Alaska Native individuals elsewhere in the US. Second, the COVID-19 pandemic changed the fielding of follow-up assessments, delayed obtaining anthropometric measures that required in-person visits, and produced loss to follow-up (due to attrition and study fielding period ending). Although participants were scheduled for study procedures during a “typical week,” presumably free of illness, it is possible that some adolescents may have had COVID-19 or other illness during the assessment, which could have affected the outcomes. The smaller sample size at follow-up may have reduced power to detect statistically significant associations.

## Conclusions

This cohort study fills a critical gap in the literature and, to our knowledge, is the first to show that specific objective and subjective sleep problems were associated with increases in mental and behavioral health symptoms and cardiometabolic risk factors among urban American Indian and Alaska Native adolescents over a 2-year follow-up period. Findings implicate the importance of developing culturally responsive interventions that target sleep as a key modifiable risk factor to promote health equity among American Indian and Alaska Native people. For example, our team is currently developing a culturally adapted sleep health intervention that integrates traditional American Indian and Alaska Native practices and values with an evidence-based behavioral sleep treatment. Findings are particularly salient considering the COVID-19 pandemic, which exacerbated health disparities. Overall, results point to the importance of identifying novel, modifiable factors to promote health equity among American Indian and Alaska Native adolescents.

## Notes

Supplement 1. **eAppendix.** Community Engagement Practices

Supplement 2.
Data Sharing Statement
