# Supplementary material for: Sleep Problems and Health Outcomes Among Urban American Indian and Alaska Native Adolescents
Source: JAMA Netw Open. 2024 Jun 4;7(6):e2414735. doi: 10.1001/jamanetworkopen.2024.14735 (PMC11151157; doi:10.1001/jamanetworkopen.2024.14735)
Supplement: Supplement 1. — eAppendix. Community Engagement Practices [file jamanetwopen-e2414735-s001.pdf]

Supplementary Online Content

Troxel WM, Klein DJ, Dong L, et al. Sleep problems and health outcomes among urban American Indian and Alaska Native adolescents. *JAMA Netw Open.* 2024;7(6):e2414735. doi:10.1001/jamanetworkopen.2024.14735

eAppendix. Community Engagement Practices

This supplementary material has been provided by the authors to give readers additional information about their work.

© 2024 Troxel WM et al. *JAMA Network Open.*

eAppendix. Community Engagement Practices

Throughout this project we engaged AI/AN community members, through our partnership with Sacred Path Indigenous Wellness Center, to ensure that community input was integrated in every stage of the research process, as our team does with all of our work with AI/ AN communities. For example, we pilot tested all materials with community members, created a study logo based on community input, hired a Native American graphic artist to create several study logos and elicited feedback from over 370 AI/AN people at partner clinics and events to determine the winning design, and hosted community meetings to address questions. This testing of materials took place over numerous meetings at organizations in both southern and central California with over 20 staff at the different organizations and at approximately 40 different members of these communities who attended presentations about the project and materials and provided feedback. Community members included parents and grandparents of teenagers and Elders. We also hired AI/AN recruiters in each community to discuss the project with families, and conducted cultural awareness training for all survey staff before they went into the field to collect data, led by study investigators, Dickerson (Inupiaq) and Johnson (Wahpeton Dakota). This cultural awareness training included topics that help to ensure cultural competence among staff as it relates to working with AI/AN people. Topics covered included learning about the impact of historical trauma among AI/AN people and issues that specifically impact this population including social inequality and discrimination. Additional topics covered included learning about the role and importance of traditional practices among AI/AN people, the diversity of AI/AN people that exist in urban areas, and the importance of living a healthy and balanced life. These topics were covered to help ensure that communication with research participants is conducted appropriately and result in garnishing the trust and comfort levels among community members.

© 2024 Troxel WM et al. *JAMA Network Open.*
