# Supplementary material for: Sleep Problems and Health Outcomes Among Urban American Indian and Alaska Native Adolescents
Source: JAMA Netw Open. 2024 Jun 4;7(6):e2414735. doi: 10.1001/jamanetworkopen.2024.14735 (PMC11151157; doi:10.1001/jamanetworkopen.2024.14735)
Supplement: Supplement 2. — Data Sharing Statement [file jamanetwopen-e2414735-s002.pdf]

Data Sharing Statement

Troxel. Sleep Problems and Health Outcomes Among Urban American Indian and Alaska Native Adolescents. *JAMA Netw Open*. Published June 04, 2024.
doi:10.1001/jamanetworkopen.2024.14735

Data
Data available: No

Additional Information
Explanation for why data not available: Our data sharing and utilization plan is guided by specific ethical concerns related to data generated by research conducted among AI/ANs and guided by prior literature and feedback from the AI/AN community and is consistent with recommendations provided in NIH's "Supplemental Information to the NIH Policy for Data Management and Sharing: Responsible Management and Sharing of American Indian/Alaska Native Participant Data". In brief, a long history exists with regard to unethical and misguided research, which has led to a mistrust of the research process within many AI/AN communities and further exploitation of this socially and economically vulnerable population. When AI/AN communities are not involved in the research process, there is significant potential for unethical and misguided research that can potentially harm AI/AN communities, particularly if there is not community-based oversight or appropriate advisory boards to ensure that research conducted utilizing this secondary data is published in an ethical and community-driven manner. As it relates to data collected under the proposed project, the data sharing plan is therefore designed to ensure that researchers utilize data in a way that will not potentially harm AI/AN communities or further stigmatize this population. Therefore, requests for data from the current study should be sent to the study PIs (Troxel, D'Amico, and Dickerson) and all requests will be reviewed and approved by the Urban Intertribal Native American Review Board.
